# *Mycoplasma pneumoniae–*Triggered Inflammatory Arthritis and Vasculitis in an Adolescent due to *Mycoplasma pneumoniae* Following an Orthopedic Procedure: A Case Report

**DOI:** 10.1155/crpe/9122947

**Published:** 2026-09-22

**Authors:** Madeleine Robertson, Gueorgui Dubrocq

**Affiliations:** ^1^ Department of Pediatrics, Baylor, Scott, and White McLane’s Children’s Hospital, Temple, Texas, USA

**Keywords:** arthritis, case report, *Mycoplasma pneumoniae*, vasculitis

## Abstract

*M*. *pneumoniae* is an atypical bacterium that commonly presents with pneumonia, but is also associated with extrapulmonary manifestations including dermatologic, CNS, gastrointestinal, rheumatologic, cardiac, and renal disease. We report a case of inflammatory arthritis with vasculitis of the lower extremities caused by *Mycoplasma pneumoniae* in a 17‐year‐old female who presented 6 weeks after left knee medial quadriceps tendon femoral ligament (MQTFL) reconstruction and patellar osteochondral allograft (OCA), with positional discoloration of her leg and clear drainage from the incision.

**Summary**
• Although *Mycoplasma* commonly presents with pulmonary manifestations, it can also present with extrapulmonary symptoms, which can include dermatologic, CNS, gastrointestinal, rheumatologic, cardiac, and renal disease.
• If *Mycoplasma pneumoniae* is suspected and the nasal swab PCR is negative, consider serology testing.
• Up to 15% of cases of *Mycoplasma* infection can be resistant to macrolides.
• Inflammatory arthritis and vasculitis are rare complications of *Mycoplasma* infection.

## 1. Case Description

A healthy 17‐year‐old female with a prior history of left knee medial quadriceps tendon femoral ligament (MQTFL) reconstruction and patellar osteochondral allograft (OCA) with an Arthrex PEEK interference screw and allograft insertion (cadaver) 6 weeks earlier was admitted with a rash and discharge from her left knee incision area.

**FIGURE 1:**
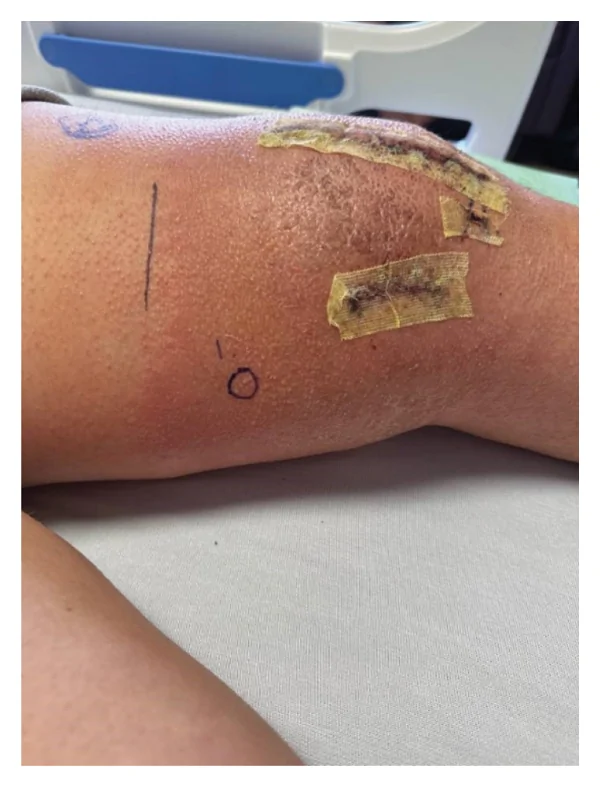
Rash on the left knee that developed 4 weeks after surgery.

**FIGURE 2:**
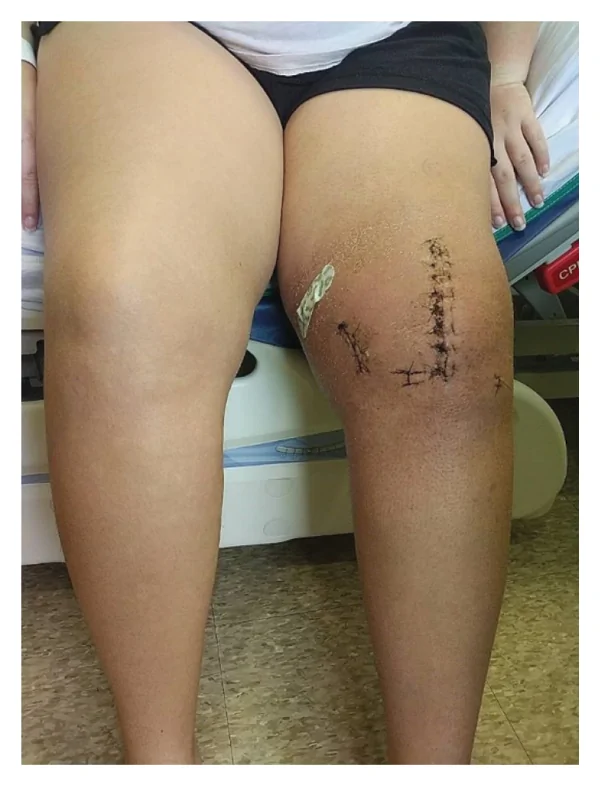
Left knee swelling and bilateral lower extremity purpura that developed after standing for several minutes.

Approximately 4 weeks following surgery, the patient developed an itchy, bumpy rash around the left knee with a small amount of clear discharge from the site of the incision. The rash was described as a red discoloration with a small amount of scale to the medial aspect of the knee, with follicular prominence and induration, with skin surrounding the knee being erythematous and warm to the touch (Figure 1). Rash was not present when supine, but while standing up for several minutes, the left knee swelled, and bilateral lower extremities became purpuric and erythematous (Figure 2). There was no functional change in her knee when the rash was present, but overall function was decreased due to postoperation status. There were no complaints of fever or systemic symptoms.

An irrigation/debridement (I&D) showed no purulence or joint effusion. Left knee wound and blood cultures were negative. Following the I&D, parenteral antibiotics (vancomycin and ceftriaxone) were started. Labs on admission showed a normal ESR, CBC, CMP, and mild elevation in CRP (15 mg/L, with a reference range of 0.0–3.2 mg/L).

MRI of the left knee did not show any signs of osteomyelitis. On Hospital day 7, the patient was transitioned to levofloxacin, as there was no clinical improvement while on vancomycin, ceftriaxone, and azithromycin. A second I&D was completed on Hospital day 10, and as there was no purulence, a decision was made to keep the Arthrex PEEK interference screw and allograft. Dermatology performed a skin biopsy on the left knee, which revealed numerous eosinophils, a nonspecific indicator of inflammation. Additional workup during admission revealed a four‐fold rise in *Mycoplasma* IgG level (1.55 U/L, positive if > 0.33 U/L), indicating recent or active infection within the last 3–6 weeks. Antinuclear antibody (ANA), antineutrophil cytoplasmic antibodies (ANCAs), interleukin‐2 (IL‐2), interleukin‐6 (IL‐6), antistreptolysin O titer (ASO), *Bartonella henselae* antibodies, and Epstein–Barr virus IgM were all negative. Evaluation for alternative causes of inflammatory arthritis such as Lyme disease, gonococcal infection, Parvovirus B19, gout, and rheumatoid arthritis was omitted during the workup due to focus on atypical pathogens.

**FIGURE 3:**
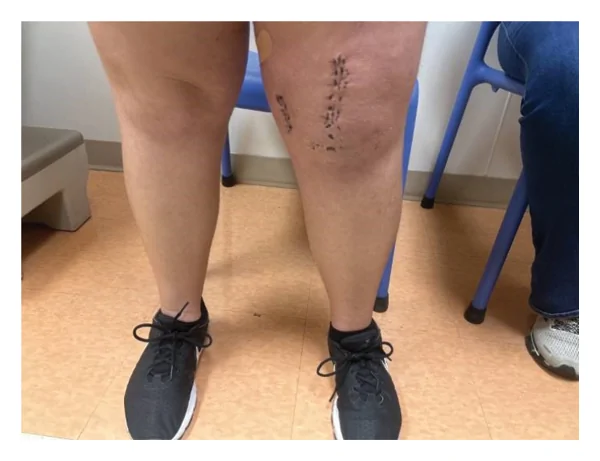
Resolution of vasculitis and a decrease in left knee swelling 3 weeks after admission.

After starting levofloxacin, the vasculitis and left knee swelling slowly resolved over a course of 2 weeks. During the follow‐up visit approximately 3 weeks from admission, the vasculitis had completely resolved, and knee swelling continued to slowly improve while completing the course of levofloxacin (Figure 3).

## 2. Discussion

*M*. *pneumoniae* is an atypical bacterium that can cause infection in children of all ages [1]. It is usually transmitted via respiratory droplets and can have an incubation period of as short as 4 days to as long as 23 days [1]. The clinical manifestation of *M*. *pneumoniae* infection is affected by the immune competence of the host [2]. Clinical symptoms present most commonly with pulmonary manifestations (pneumonia and prolonged cough), but *M*. *pneumoniae* causes a wide range of extrapulmonary symptoms, which can include dermatologic, CNS, gastrointestinal, rheumatologic, cardiac, and renal disease [3]. Reactive arthritis is the least common extrapulmonary manifestation, and incidence is thought to be between 0.9% and 2.1% [4]. There are 3 suspected patterns of joint involvement: the first occurring during acute infection and is categorized as migratory, polyarticular, and mainly affecting large joints. The second occurs after the acute phase of infection, mostly commonly with stiffness, hyperemia, and edema of several joints [5]. The third occurs in patients with hypogammaglobulinemia and commonly results in septic arthritis [6].

This case presents a teenage girl with inflammatory arthritis and vasculitis in the setting of *Mycoplasma pneumoniae* after acute infection. In 2024, there has been an outbreak of *Mycoplasma pneumoniae* in the United States. According to the CDC, up to 15% of *Mycoplasma pneumoniae* are resistant to macrolides, such as azithromycin. In the literature, it is reported that 20% are resistant [7]. In many cases, there is a delay in the diagnosis of *Mycoplasma pneumoniae* if the nasal swab PCR is negative, as the serologies take time to give the result, and commercial microbiology labs cannot identify it via culture methods [8]. There is limited knowledge, and few cases in the literature of reactive arthritis and *Mycoplasma pneumoniae* are reported in children. In a study looking at causes of arthritis in children by Kahn, 25% (18 patients) of children were found to have septic arthritis, and 73% (53 patients) were found to have nonseptic arthritis. When testing via PCR was completed on patients with nonpyogenic arthritis, 33% were found to have positive *M*. *pneumoniae* IgG [9]. In another study looking at the presence of *Mycoplasma* in synovial fluid of patients with joint disorders, *Mycoplasma pneumoniae* was not found in joint fluid samples but was thought to be a cofactor in triggering reactive inflammatory joint disease [10].

An association between vasculitis and *Mycoplasma pneumoniae* is rare in the literature. In a literature review about vasculitis as an extrapulmonary manifestation of *Mycoplasma*, only 6 cases had been reported, and only 3 of which were pediatric cases [11]. However, this can be a challenging diagnosis, as in vasculitis, the respiratory viral panel is often negative, and most cases are diagnosed via antibody testing.

In our case, the diagnosis was made based on a fourfold increase in *Mycoplasma pneumoniae* IgG titer, while IgM antibody was negative. Without a positive PCR result, this introduces diagnostic uncertainty, as it can reflect a secondary event. Although our case strongly suggests recent *Mycoplasma pneumoniae* exposure based on clinical history and serology results, without confirmatory PCR testing, a definitive diagnosis is challenging. This case emphasizes and highlights the need for clinicians to recognize inflammatory arthritis and vasculitis as complications of *Mycoplasma pneumoniae*. Knowledge of this potential association may allow clinicians to consider a diagnosis of *Mycoplasma pneumoniae* earlier in the clinical presentation, and it may help avoid unnecessary procedures. The case also stresses that if a patient with *Mycoplasma* infection is not clinically improving while on azithromycin, the clinician should consider transitioning to levofloxacin or doxycycline.

### 2.1. Limitations

This report has limitations. Testing relied solely on *Mycoplasma* antibody detection (serology), which was performed late after the onset of disease, and without direct pathogen identification via PCR. Treatment was guided by the serology results and clinical course. Ideally. Serology, along with commercial PCR tests, increases the reliability and accuracy of diagnosis of *Mycoplasma*.

## Funding

No funding was received for this case report.

## Ethics Statement

Ethical approval was waived by the ethics review committee of the hospital because this was an anonymous case presentation.

## Consent

Parents of the patient have provided permission to publish the case.

## Conflicts of Interest

The authors declare no conflicts of interest.

## Data Availability

Data sharing is not applicable to this article as no datasets were generated or analyzed during the current study.
